# Rice *miR172* induces flowering by suppressing *OsIDS1* and *SNB*, two AP2 genes that negatively regulate expression of *Ehd1* and florigens

**DOI:** 10.1186/s12284-014-0031-4

**Published:** 2014-11-19

**Authors:** Yang-Seok Lee, Dong-Yeon Lee, Lae-Hyeon Cho, Gynheung An

**Affiliations:** Crop Biotech Institute & Department of Plant Systems Biotech, Kyung Hee University, Yongin, 446-701 Korea

**Keywords:** AP2 family, Floral transition, miR172, Phytochromes, Rice

## Abstract

**Background:**

Rice is a facultative short-day plant that flowers under long days (LD) after a lengthy vegetative phase. Although several inhibitors that delay flowering have been identified, the process by which rice eventually flowers under non-permissive LD conditions is not well understood.

**Results:**

Overexpression of *miR172* reduced flowering time significantly, suggesting its role as an inducer. Levels of *miR172* increased as plants aged, further supporting our findings. Transcripts of *SNB* and *OsIDS1*, two members of the AP2 family that have the *miR172* target site, were reduced in older plants as the level of *miR172* rose. Overexpression of those AP2 genes delayed flowering; overexpression of *miR172*-resistant forms of *SNB* or *OsIDS1* further delayed this process. This demonstrated that the AP2 genes function downstream of *miR172*. Two florigen genes -- *Hd3a* and *RFT1 --* and their immediate upstream regulator *Ehd1* were suppressed in the AP2 overexpression plants. This suggested that the AP2 genes are upstream repressors of *Ehd1*. In phytochrome mutants, *miR172d* levels were increased whereas those of *SNB* and *OsIDS1* were decreased. Thus, it appears that phytochromes inhibit *miR172d*, an AP2 suppresser.

**Conclusions:**

We revealed that *miR172d* developmentally induced flowering via repressing *OsIDS1* and *SNB*, which suppressed *Ehd1*. We also showed that phytochromes negatively regulated *miR172*.

**Electronic supplementary material:**

The online version of this article (doi:10.1186/s12284-014-0031-4) contains supplementary material, which is available to authorized users.

## Background

Rice flowers earlier under short day (SD) conditions than under long days (LD). The photoperiodic flowering pathway of rice is controlled by *OsGIGANTEA* (*OsGI*) (Yano et al. [2000]; Hayama et al. [2002], [2003]; Kojima et al. [2002]; Doi et al. [2004]). This gene regulates flowering time by promoting *Heading date 1* (*Hd1*), an ortholog of *Arabidopsis CONSTANS* (*CO*) (Yano et al. [2000]; Hayama et al. [2002], [2003]). *Hd1* enhances *Early heading date 1* (*Ehd1*) expression under SD but inhibits its expression under LD (Wei et al. [2010]; Ishikawa et al. [2011]). *Ehd1* is an immediate upstream positive regulator of *Heading date 3a* (*Hd3a*) and *Rice Flowering Locus T 1* (*RFT1*), which encode florigens that promote flowering (Doi et al. [2004]; Tamaki et al. [2007]; Komiya et al. [2008], [2009]).

Because *Ehd1* is modulated by several regulators, it acts as a mediator of various floral signals. For example, *Early heading date 2* (*Ehd2*)/*OsINDETERMINATE 1* (*OsId1*)/*Rice INDETERMINATE 1* (*RID1*) is a constitutive activator of *Ehd1* (Matsubara et al. [2008]; Park et al. [2008]; Wu et al. [2008]). Loss-of-function mutants in the gene do not flower under either SD or LD. *OsMADS51* also acts as a positive regulator of *Ehd1*, specifically under SD (Kim et al. [2007]). One major repressor of *Ehd1* is *Grain yield and heading date 7* (*Ghd7*), which functions preferentially under LD (Xue et al. [2008]). Most early-flowering rice cultivars show a disruption in *Ghd7* expression (Xue et al. [2008]). A chromatin remodeling factor, *OsTrithorax 1* (*OsTrx1*), suppresses *Ghd7* by binding to *Early heading date 3* (*Ehd3*) (Matsubara et al. [2011]; Choi et al. [2014]). In addition, *OsLFL1* inhibits *Ehd1* when over-expressed (Peng et al. [2007], [2008]). However, expression of the former is suppressed by another chromatin remodeling factor, *OsVIN3-like 2* (*OsVIL2*), when binding to the PRC2 complex (Yang et al. [2013]). *OsMADS50* induces *Ehd1* by blocking *OsLFL1* and *Ghd7* (Lee et al. [2004]; Ryu et al. [2009]; Choi et al. [2014]). A third gene, *OsCOL4*, constitutively inhibits flowering time by suppressing *Ehd1* (Lee et al. [2010]). Overexpression of the former delays flowering whereas knockout mutations cause early flowering. Finally, *DTH8* and *Hd16* preferentially suppress flowering time under LD by inhibiting *Ehd1* (Xue et al. [2008]; Wei et al. [2010]; Hori et al. [2013]).

Micro RNAs inhibit expression of target genes by cleaving mRNA or translational suppression (Jones-Rhoades et al. [2006]; Voinnet [2009]). *miR172* and *miR156* are involved in phase transition (Aukerman and Sakai [2003]; Lauter et al. [2005]; Wu and Poethig [2006]; Poethig [2009]). *miR156* plays roles in early vegetative stages, while *miR172* functions later stages of develop (Aukerman and Sakai [2003]; Lauter et al. [2005]; Wu and Poethig [2006]; Chuck et al. [2007]; Poethig [2009]). In *Arabidopsis*, *miR156* targets 10 members (*SPL2*, *SPL3*, *SPL4*, *SPL5*, *SPL6*, *SPL9*, *SPL10*, *SPL11*, *SPL13*, and *SPL15*) of *SQUAMOSA PROMOTER BINDING PROTEIN-LIKE* (*SPL*) family. *SPL9* prompts *miR172* expression and the other *SPL* genes redundantly function in regulating *miR172*. The role of *miR172* in controlling flowering time has been reported for *Arabidopsis*, maize, barley, and soybean (Aukerman and Sakai [2003]; Chen [2004]; Lauter et al. [2005]; Jung et al. [2007]; Mathieu et al. [2009]; Nair et al. [2010]; Yoshikawa et al. [2013]). In *Arabidopsis* and maize, its temporal expression increases gradually as plants age (Aukerman and Sakai [2003]; Lauter et al. [2005]). In rice, *miR172* is most highly expressed during later vegetative stages and in developing panicles (Zhu et al. [2009]; Lee and An [2012]).

AP2 family genes are involved in various processes, including floral organ identity, shattering, and flowering time (Aukerman and Sakai [2003]; Chen [2004]; Lauter et al. [2005]; Lee et al. [2007]; Jung et al. [2007]; Chuck et al. [2008]; Mathieu et al. [2009]; Zhu et al. [2009]; Lee and An [2012]; Zhou et al. [2012]; Yoshikawa et al. [2013]). Six *Arabidopsis* genes in this family -- *APETALA 2* (*AP2*), *TARGET OF EAT 1*(*TOE1*), *TOE2*, *TOE3*, *SCHLAFMUTZE* (*SMZ*), and *SCHNARCHZAPFEN* (*SNZ*) -- delay flowering in an age-dependent manner (Park et al. [2002]; Aukerman and Sakai [2003]; Schmid et al. [2003]; Chen [2004]; Jung et al. [2007]; Mathieu et al. [2009]), and are suppressed by *miR172* (Schmid et al. [2003]; Kasschau et al. [2003]; Chen [2004]; Schwab et al. [2005]; J*u* ng et al. [2007]; Mathieu et al. [2009]). In maize, enhancement of *GLOSSY15* (*GL15*), an *AP2* member, delays phase transition from the vegetative to the reproductive stage (Lauter et al. [2005], Zhu and Helliwell [2011]). Its temporal expression gradually decreases as plants mature, and this gene is also down-regulated by *miR172* (Lauter et al. [2005]). In rice, five AP2-like genes (*SNB*, *OsIDS1*, *SHAT1*, *Os05g03040*, and *Os06g43220*) contain the *miR172* target sites (Sunkar et al. [2005]; Zhu et al. [2009]). While *SNB* and *OsIDS1* control floral organ identity and spikelet development, *SHAT1* is involved in seed shattering (Sunkar et al. [2005]; Lee et al. [2007]; Zhu et al. [2009]; Lee and An [2012]; Zhou et al. [2012]).

Although an antagonistic role for *miR172* and AP2 genes in floral transition has been described in *Arabidopsis* and maize, their functions in rice have not been reported. Here, we demonstrated in rice that *miR172* induces flowering time by suppressing two *AP2* family members - *SNB* and *OsIDS1* - that are negative regulators of *Ehd1*.

## Results

### Temporal expression patterns of miR172s and AP2 genes are antagonistic

**Figure 1:**
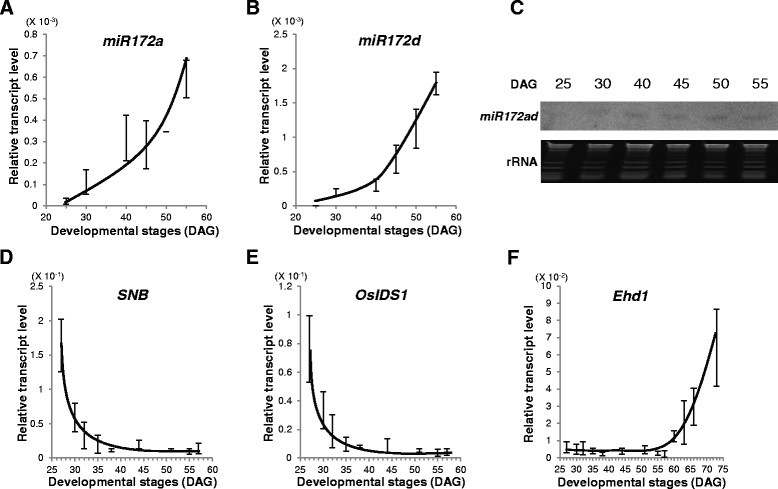
**Temporal expression patterns of** ***miR172*** **and AP2 genes. (A, B)** RT-PCR analysis of pri-*miR172a* **(A)** and pri-*miR172d* **(B)** in WT plants under LD. Y-axis, relative transcript level compared with *OsUbi1*. Error bars indicate standard deviations; *n* = 4 or more. Middle regions of fully emerged uppermost leaves were sampled at 30, 40, 45, 50, 55, and 60 DAG. **(C)** Northern blot analysis of mature *miR172* (top). Ethidium bromide-stained ribosomal RNA served as loading control (bottom). Middle regions were used from leaves sampled at 25, 30, 40, 45, 50, and 55 DAG. **(D, E)** RT-PCR analysis of transcript levels of *SNB* **(E)** and *OsIDS1* **(E)** under LD. Middle regions were used from leaves sampled at 27, 30, 32, 35, 38, 44, 51, 55, and 57 DAG. **(F)** RT-PCR analysis of *Ehd1* transcripts under LD, using leaves sampled at 27, 30, 32, 35, 38, 44, 51, 55, 57, 60, 63, 66, and 73 DAG. Expression was monitored at ZT 18 h for *miR172* and at ZT 2 h for AP2 genes and *Ehd1*. Y-axis, relative transcript level compared with *OsUbi1*. Error bars indicate standard deviations; n = 4 or more.

To examine whether miR172 levels change as plants develop, we monitored the temporal expressions of *miR172a* and *miR172d* in leaf blades. The other two *miR172* members in rice - *miR172b* and *miR172c* – were not measured because they are expressed mainly in panicles and roots, respectively, and are not likely involved in controlling flowering time (Jeong et al. [2011]). Levels of pri-*miR172a* and pri-*miR172d* increased gradually as plants aged (Figure 1A and B). To verify this, we conducted northern blot analysis of mature *miR172.* Because the sequences of mature *miR172a* and *miR172d* are identical, we were only able to measure the total amount of both micro RNAs. This analysis confirmed that mature *miR172ad* levels were gradually increased (Figure 1C).

The *miR172* s target several AP2 genes, with temporal expression of the latter type being slowly diminished in *Arabidopsis* and maize (Aukerman and Sakai [2003]; Lauter et al. [2005]; Jung et al. [2007]; Mathieu et al. [2009]; Zhu and Helliwell [2011]). In all, six AP2 genes have *miR172* target sites (Zhu and Helliwell [2011]). Other miRNAs and their targeted genes also show antagonistic expression patterns (Jeong and Green [2013]; Jung et al. [2013]). We examined the temporal expression of two rice AP2 genes in leaf blades: *SNB* and *OsIDS1*. As expected, their transcript levels were high at younger developmental stages but rapidly declined to a low level at 35 days after germination (DAG) (Figure 1D and E). *Ehd1* mRNA expression began to increase at 57 DAG (Figure 1F). Under SD, transcript levels of the AP2 genes dropped rapidly at 18 DAG, immediately before the level of *Ehd1* began to rise (Additional file 1: Figure S1). This suggested that the AP2 genes are likely targets of *miR172a* and *miR172d*.

### Overexpression of SNB and OsIDScauses late flowering

**Figure 2:**
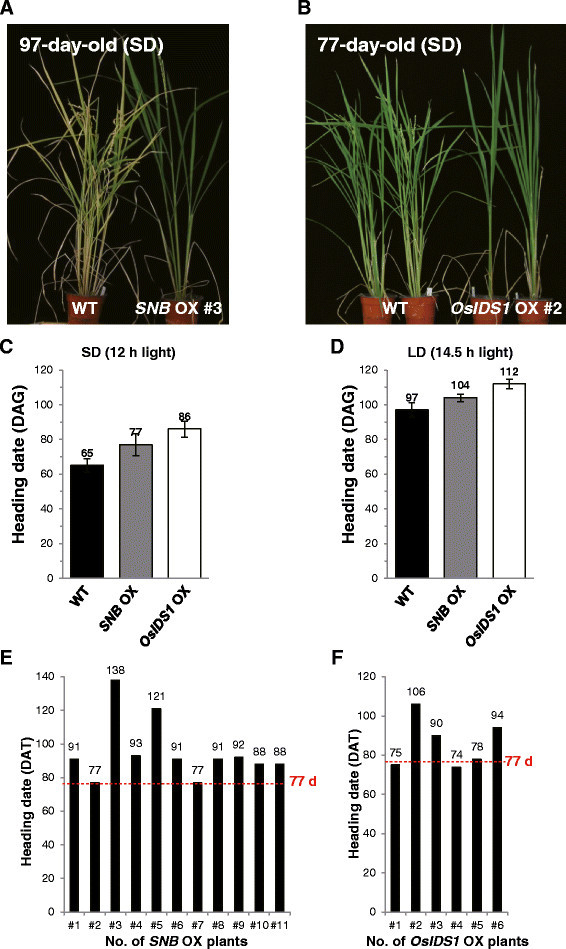
**Analysis of** ***SNB*** **and** ***OsIDS1*** **overexpression plants. (A)** Phenotypes of *SNB* overexpression plant (*SNB* OX #3) at 97 DAG when grown under SD. **(B)** Phenotypes of *OsIDS1* overexpression plants (*OsIDS1* OX #2) at 77 DAG when grown under SD. **(C, D)** Heading dates for *OsIDS1* and *SNB* OX plants under SD **(C)** or LD **(D)**. Days-to-heading was scored when first panicle bolted. Error bars indicate standard deviations; *n* = 10-20 plants. **(E, F)** Flowering time for *SNB* (E) and *OsIDS1* (Ff) OX plants (T1 generation) in greenhouse. Days-to-heading was scored when first panicle bolted.

To examine the functional roles of AP2 genes in flowering time, we generated transgenic rice plants that over-express *SNB* and *OsIDS1* (Additional file 1: Figure S2). Such overexpression (OX) of *miR172*-targeted AP2 causes late-flowering phenotypes in *Arabidopsis* (Jung et al. [2007]; Mathieu et al. [2009]). Similarly, we noted that *SNB* OX and *OsIDS1* OX plants flowered late when grown in the greenhouse (Figure 2A and B). Among our 11 *SNB* OX transgenics, flowering was delayed by 2 to 8 weeks for nine of them (Figure 2E). Flowering time for the *SNB* OX plants was correlated with the degree of *SNB* expression (Additional file 1: Figure S2A, S2B, and S2C). Likewise, *OsIDS1* OX plants flowered 3 to 5 weeks later than usual (Figure 2F). Their heading date was correlated with amounts of *OsIDS1* transcript (Additional file 1: Figure S2D, S2E, and S2F). Three *SNB* OX plants (#3, 4, and 5) and three *OsIDS1* OX plants (#2, 3, and 6) were selected for further analysis. In studying photoperiod dependency of those genes, we found that flowering time for *SNB* OX #3 was delayed by about 12 d under SD and about 7 d under LD (Figure 2A, C, and D). Similarly, *OsIDS1* OX #2 flowered 3 weeks later than usual under SD and 2 weeks later under LD (Figure 2B, C, and D). These observations demonstrated that *SNB* and *OsIDS1* function as flowering repressors in rice.

### SNB and OsIDS1 repress the floral transition by inhibiting Ehd1

**Figure 3:**
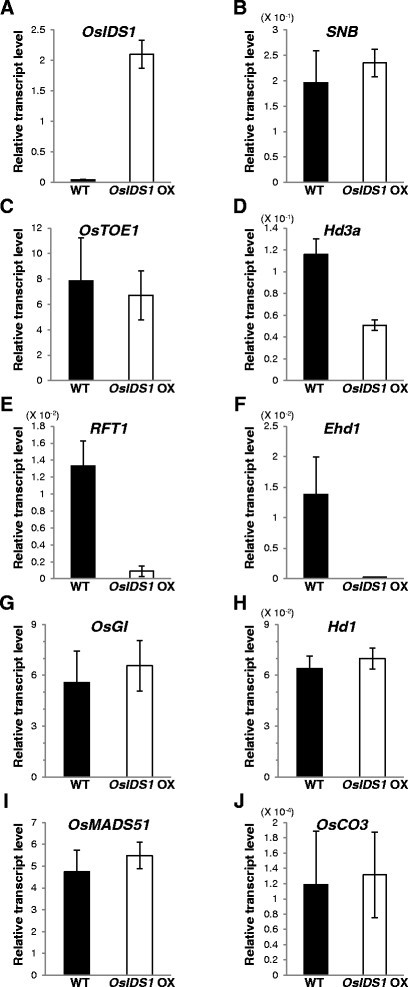
**Transcript levels of floral regulators in the** ***OsIDS1*** **OX.** Transcript levels of *OsIDS1* **(A)**, *SNB* **(B)**, *OsTOE1* **(C),** *Hd3a* **(D)**, *RFT1* **(E)**, *Ehd1* **(F)**, *OsGI* **(G)**, *Hd1* **(H)**, *OsMADS51* **(I)**, and *OsCO3* **(J)** in WT (closed boxes) and *OsIDS1* OX plants (open boxes) under SD. Middle regions of fully emerged uppermost leaves were sampled at 30 DAG. For *OsGI* and *Hd1*, monitoring occurred at ZT 10 h and ZT 14 h, respectively; for others, at ZT 2 h. Y-axis, relative transcript level compared with rice *OsUbi1*. Error bars indicate standard deviations; *n* = 4 or more.

To elucidate the roles of *SNB* and *OsIDS1* in controlling flowering time, we measured transcript levels of the previously identified floral regulators in OX plants. Because the delayed-flowering phenotype was more severe in *OsIDS1* OX #2, we analyzed those plants. Increased expression of *OsIDS1* did not affect transcript levels of the AP2 genes (Figure 3B and C). Transcripts of *Hd3a* and *RFT1*, two florigens in rice, were significantly reduced in those plants (Figure 3D and E), as were transcripts of *Ehd1* (Figure 3F). However, levels of other flowering activators, i.e., *OsGI*, *Hd1*, *OsMADS51*, and *OsCO3*, were not altered in the OX plants (Figure 3G, H, I, and J). Overexpression of *OsIDS1* also had no influence on flowering time regulators such as *OsPhyB*, *OsMADS50*, *Ghd7*, *OsId1*, *OsCOL4*, *OsMADS56*, *Ehd3*, and *OsTrx1* (Additional file 1: Figure S3). In the *SNB* OX #3, transcript levels of *Ehd1*, *Hd3a*, and *RFT1* were also decreased, but those of *OsGI*, *Hd1*, *OsMADS51*, and *OsCOL4* were not changed (Additional file 1: Figure S4). These observations indicated that *SNB* and *OsIDS1* repress the floral transition by inhibiting the expression of *Ehd1*.

**Figure 4:**
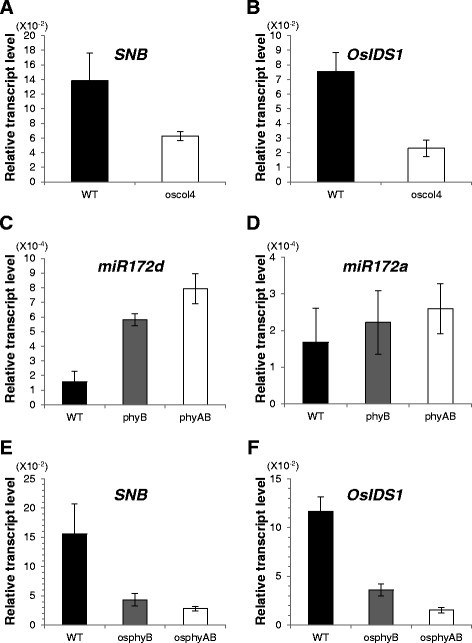
**Expression of** ***miR172*** **s and AP2 genes in** ***oscol4*** **,** ***osphyB*** **, and** ***osphyA osphyB*** **mutants. (A, B)** Transcript levels for *SNB* **(A)** and *OsIDS1* **(B)** in WT (black bars) and *oscol4* (open bars) plants under LD. **(C, D)** Transcript levels for *miR172d* **(C)** and *miR172a* **(D)** in WT (black bars), *osphyB* (gray bars), and *osphyA osphyB* (open bars) plants under LD. **(E, F)** Transcript levels for *SNB* **(E)** and *OsIDS1* **(F)** in WT (black bars), *osphyB* (gray bars), and *osphyA osphyB* (open bars) plants under LD. Middle regions of fully emerged uppermost leaves were sampled at 35 DAG. Expression was monitored at ZT 11 h for *miR172* s and at ZT 2 h for *SNB* and *OsIDS1*. Y-axis, relative transcript level compared with *OsUbi1*. Error bars indicate standard deviations; *n* = 4 or more.

Levels of *SNB* and *OsIDS1* did not differ between `Dongjin′ and `Kitaake′ rice. The latter is an early-flowering cultivar and carries mutations in *Ghd7* and *PRR37* (Kim et al. [2013]). Therefore, this demonstrated that the AP2 genes are not likely influenced by Ghd7 and PRR37. In addition, AP2 expression was not significantly altered in mutants defective in regulatory genes such as *Ghd7*, *OsGI*, *Ehd1*, *OsMADS50*, *OsMADS51*, *OsId1*, *OsTrx1*, and *OsVIL2*, all of which control flowering time (Additional file 1: Figure S5). However, expression of *SNB* and *OsIDS1* was affected in the *oscol4* and *hd1* mutants (Figure 4A and B; Additional file 1: Figure S5A). This suggested that expression of AP2 genes is positively controlled by *OsCOL4* and *Hd1*.

### miR172d induces flowering time by suppressing AP2 genes

**Figure 5:**
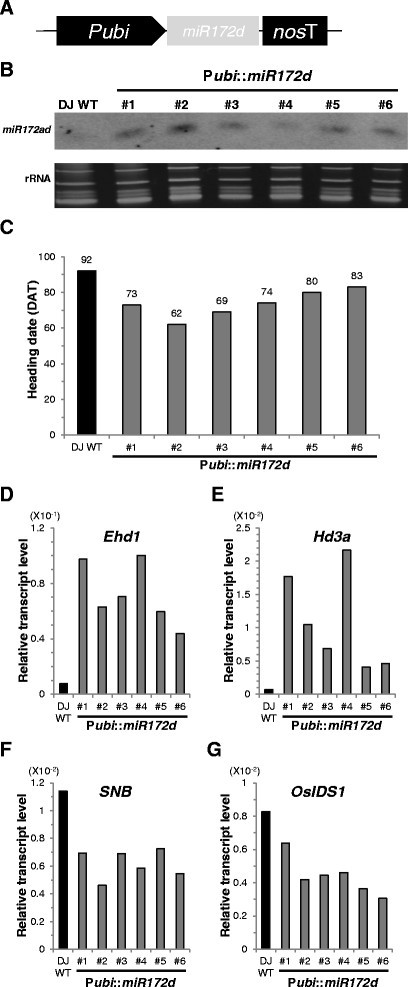
**Phenotypes of** ***miR172d*** **OX plants. (A)** Scheme of *miR172d* OX construct. Spanning region of *miR172d* was sub-cloned into pGA1611 vector between maize *ubiquitin* promoter (P*ubi*) and *nopaline synthase* terminator (*nos* T). **(B)** Northern blot analysis of expression levels of mature *miR172* in WT and *miR172d* OX plants (top). Ethidium bromide-stained ribosomal RNA was used as loading control (bottom). **(C)** Flowering time for 6 *miR172d* OX plants compared with WT when grown in greenhouse. Days-to-heading was scored when first panicle bolted. **(D-G)** Expression levels of *Ehd1* **(D)**, *Hd3a* **(E)**, *SNB* **(F)**, and *OsIDS1* **(G)** in WT and *miR172d* OX plants.

The transcripts of *SNB* and *OsIDS1* carry a *miR172* target site (Lee and An [2012]). To examine whether *miR172* controls flowering time, we generated six transgenic rice plants that over-express *miR172d* (Figure 5A). In all plants, transcripts were increased (Figure 5B) and the time to flowering was shortened by 9 to 30 d (Figure 5C). Levels of flowering activators *Ehd1* and *Hd3a* were also substantially higher in the *miR172d* OX lines (Figure 5D and E) while expression of *SNB* and *OsIDS1* was decreased in those lines (Figure 5F and G). These results suggested that *miR172d* induces flowering by reducing AP2 gene expression.

**Figure 6:**
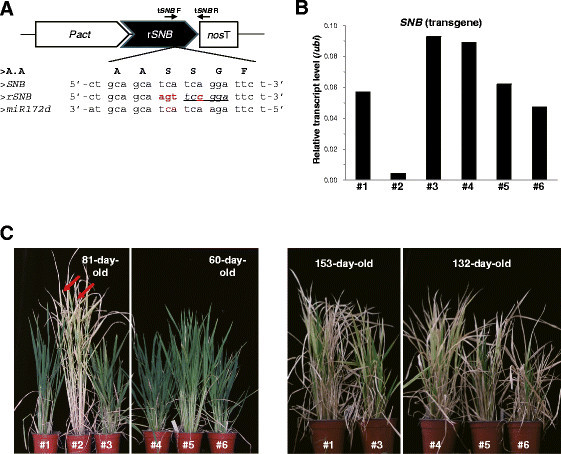
**Phenotypes of** ***miR172*** **-resistant** ***SNB*** **OX plants. (A)** Scheme of *miR172*-resistant *SNB* (r*SNB*) construct. Four synonymous mutations (red characters) were introduced into *miR172* target sequences at C-terminus of *SNB* full-length cDNA. Primers t*SNB* F and R were used. Underlined sequence (*tccgga*) indicates restriction enzyme site by *ACC* III. P*act*, *actin* promoter; *nos* T, *nopaline synthase* terminator. **(B)** Expression level of *SNB* in r*SNB* OX plants. **(C)** Phenotypes of r*SNB* OX plants grown under SD. Bolted panicles are shown by red arrows.

To confirm that *miR172* controls flowering time via the AP2 genes, we constructed the *miR172*-resistant form of *SNB* (r*SNB*) by changing the *miR172* target site CTGCAGCATCATCAGGATTCT to CTGCAGCAATGTCCGGATTCT (Figure 6A). Of the six transgenic rice plants carrying this r*SNB* construct, five lines (#1, 3, 4, 5, and 6) expressed the transgene at substantially high levels (Figure 6B). The r*SNB* transcript can be distinguished from the endogenous *SNB* transcript due to the restriction enzyme site *ACC* III that is generated in the r*SNB* OX construct. Here, the RT-PCR products of those r*SNB* OX plants were digested with *ACC* III, supporting our findings that most of the *SNB* transcripts in the OX plants were r*SNB* (Additional file 1: Figure S7). These r*SNB* OX plants eventually died without having flowered, even after several months of growth (Figure 6C). Moreover, transgenic plants over-expressing the *miR172*-resistant form of *OsIDS1* (r*OsIDS1*) did not produce any flowers for more than one year (Additional file 1: Figure S6). These observations further demonstrated that *miR172* s induce flowering time by suppressing AP2 transcripts in rice.

### The late-flowering phenotype of rSNB OX plants is rescued by overexpression of Ehd1

**Figure 7:**
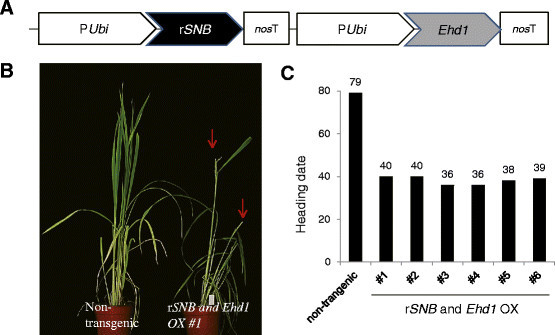
**Phenotype of transgenic plant overexpressing both r** ***SNB*** **and** ***Ehd1.*** **(A)** Diagram of *miR172*-resistant *SNB* (r*SNB*) and *Ehd1* double OX construct. p*Ubi*, maize *ubiquitin* promoter; *nos* T, *nos* terminator. **(B)** Phenotypes of r*SNB* and *Ehd1* OX #1 plants. Photograph was taken at 42 days after transplanting. Red arrows indicate emerged panicles. **(C)** Heading date of r*SNB* and *Ehd1* OX primary transgenic plants grown in greenhouse.

Because *Ehd1* transcripts were reduced in *SNB* OX plants, we postulated that the AP2 gene functions upstream of *Ehd1*. To confirm this hypothesis genetically, we generated transgenic plants over-expressing both r*SNB* and *Ehd1* (Figure 7A). It was previously reported that overexpression of *Ehd1* causes early flowering (Osugi et al. [2011]), and we also observed early-flowering phenotypes of *Ehd1* OX plants (Additional file 1: Figure S8). Thus, if *SNB* functions downstream of *Ehd1*, we would expect that transgenic plants expressing both genes do not flower early. Instead, those plants did flower early and their morphology was similar to plants that over-express only *Ehd1* (Figure 7B and C). This observation supports that *SNB* inhibits flowering time by suppressing *Ehd1*.

### miR172a and miR172d are negatively regulated by phytochromes in rice

**Figure 8:**
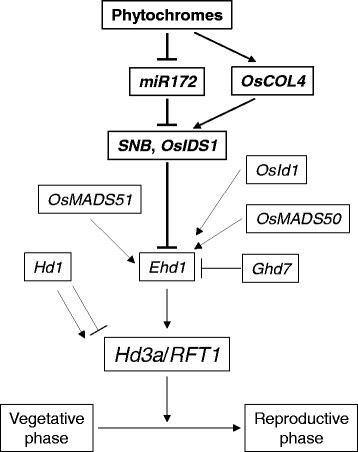
**Pathway for control of flowering time via** ***miR172d.*** Phytochromes are negative regulators of *miR172d* that suppress AP2 genes *SNB* and *OsIDS1*, both of which are negative regulators of *Ehd1*.

The level of *miR172* is increased in *phyB* mutants of *Arabidopsis* (Jung et al. [2007]). To study whether *miR172* in rice is also regulated by phytochrome, we measured the levels of pri-*miR172a* and pri-*miR172d* in mutants (Figure 4). This analysis revealed that pri-*miR172a* and pri-*miR172d* transcripts were elevated in the *osphyB* mutant and further increased in the *osphyA osphyB* double mutant (Figure 4C and D). This indicated that *miR172a* and *miR172d* are negatively regulated by phytochrome. Transcript levels of *SNB* and *OsIDS1* were also significantly reduced in the *osphyB* single and the *osphyA osphyB* double mutants (Figure 4E and F). These observations suggested that phytochromes can inhibit flowering time by suppressing *miR172a* and *miR172d*, which interfere with *SNB* and *OsIDS1* expression (Figure 8).

## Discussion

We have demonstrated that *miR172d* induces flowering by suppressing *SNB* and *OsIDS1*. Overexpression of the AP2 genes delays flowering more significantly under SD, the permissive condition. This phenotype is similar to that of *Arabidopsis SMZ-* OX plants (Mathieu et al. [2009]). Overexpression of *SMZ* causes a significant delay in flowers under LD, the permissive condition for that species, but not under SD (Mathieu et al. [2009]). These reports provide evidence that AP2 genes preferentially block the floral transition under inductive conditions.

Although flowering is inhibited under LD, rice plants do flower eventually under normally suppressive conditions, albeit after a certain period of vegetative growth. We showed here that *miR172d* levels were increased in the leaves as a plant aged and that flowering was induced when that gene was over-expressed. This indicated that the microRNA plays a role in the developmental control of flowering time in rice, as also observed from *Arabidopsis* (Jung et al. [2007]).

*SNB* and *OsIDS1* are flowering repressors that are highly expressed in young leaves. However, their expression gradually declined to minimal levels at 35 DAG when *miR172* transcripts started to increase. Expression of the florigens and *Ehd1* began at 60 DAG while that of the AP2 genes remained low. This indicated that the latter are major suppressors of the downstream floral signals.

*Ghd7* also functions to repress *Ehd1*, but its transcription peaks at 2 to 3 weeks before declining to a low level. This occurs much earlier than when the florigen genes are expressed (Matsubara et al. [2011]; Kim et al. [2013]). *OsCOL4* is another regulator that suppresses *Ehd1* and the florigen genes. In particular, *OsCOL4* expression is maintained at a high level in the early vegetative stages but decreases when *Ehd1* expression begins (Lee et al. [2010]). This temporal expression pattern is similar to *SNB* and *OsIDS1* except that *OsCOL4* is reduced later than the AP2 genes. Therefore, these results suggest that the AP2 genes as well as *Ghd7* and *OsCOL4* coordinately suppress flowering time.

Except in *oscol4* and *hd1* plants, *AP2* expression is not significantly altered in most flowering mutants, thereby implying that those genes are controlled by *OsCOL4* and *Hd1*, but not by other flowering time regulators. We have previously reported that OsCOL4 is a constitutive repressor of *Ehd1* (Lee et al. [2010]). Because this is also true of AP2 genes, it is likely that OsCOL4 suppresses *Ehd1* by inducing AP2 expression. Hd1 also suppresses flowering time via repressing *Hd3a* and *RFT1* under LD conditions (Yano et al. [2000]; Hayama et al. [2003]). These indicate that the AP2 genes and *OsCOL4* co-operatively suppress flowering time.

We observed that *miR172d* expression is negatively affected by phytochrome activity. Considering that AP2 genes are controlled by miR172, we might conclude that phytochromes support vegetative growth by maintaining AP2 expression. Both *Ghd7* and *OsCOL4* are positively modulated by *OsPhyB* (Lee et al. [2010]; Osugi et al. [2011]). Therefore, it is apparent that phytochromes influence these flowering regulators collectively.

In plants, the *miR172*/*AP2* module is inversely correlated with the *miR156*/SPL module (Aukerman and Sakai [2003]; [2005]; Wu and Poethig [2006]; Chuck et al. [2007]; Poethig [2009]). In rice, *miR156* genes are predominantly expressed in the young shoots, etiolated shoots, and seedling leaves (Xie et al. [2006]) and its target *OsSPL* genes are expressed high in young panicles (Xie et al. [2006]). Overexpression of *miR156* OX causes various phenotypes such as increase of tiller numbers, late flowering, dwarfism, and decrease of spikelet numbers (Xie et al. [2006]). In maize, overexpression of the maize *miR156* prevents flowering and increases starch content (Chuck et al. [2007]; [2011]). This late-flowering phenotype is similar to that of *SNB* OX or *OsIDS1* OX plants. Overexpression of the maize *miR156* also causes increase of biomass and tiller number in maize and also in *Arabidopsis*, *Brachypodium* and switchgrass (Chuck et al. [2011]). These phenotypes are similar to those of *OsmiR156* OX (Xie et al. [2006]; Chuck et al. [2011]).

## Conclusions

We demonstrated that over-expressions of *OsIDS1* and *SNB* inhibited flowering time in rice by suppressing expression of *Ehd1*. In addition, expressions of the AP2 genes were repressed by *miR172* and the later was increased in the *osphyB* and *osphyA osphyB* mutants. Based on these findings, we concluded that *miR172* induced flowering by suppressing the AP2 genes and the microRNA gene was inhibited by phytochromes. The facultative LD-flowering phenotype of rice can be explained in part by the miR172-AP2 pathway.

## Methods

### Plant material and growth conditions

We have previously developed T-DNA-tagging lines in *Oryza sativa* japonica cv. Dongjin (Jeon et al. [2000]). The flanking sequences were determined via inverse PCR (An et al. [2003]; Ryu et al. [2004]). T-DNA insertional mutants *osphyA-2*, *osphyB-2*, *osmads50-1*, *osmads51-1*, *hd1-1*, *oscol4-2*, *osvil2-1*, and *ostrx1-1* were reported earlier (Lee et al. [2004]; Jeong et al. [2007]; Kim et al. [2007]; Ryu et al. [2009]; Lee et al. [2010]; Yang et al. [2013]; Choi et al. [2014]). RNAi-suppressed plants of *Ehd1* RNAi and *OsId1* RNAi have been described previously (Kim et al. [2007]; Park et al. [2008]). A near isogenic line (NIL) that carries the *ghd7* allele was presented by Kim et al. ([2013]). Seeds were germinated on a half-strength Murashige and Skoog medium containing 3% sucrose. They were incubated for one week at 28°C under constitutive light as previously reported (Yi and An [2013]). Seedlings were transplanted into soil and cultured in growth chambers under either SD (12 h light at 28°C, humidity 70%; 12 h dark at 25°C, humidity 50%) or LD (14.5 h light at 28°C, humidity 70%; 9.5 h dark at 25°C, humidity 50%) as previously reported (Yi and An [2013]).

### Construction of *osphyA osphyB* double mutants

The *osphyA osphyB* double mutants were obtained by crossing *osphyA-2* and *osphyB-2* single mutants. This *osphyA-2* mutant is a null allele generated by a T-DNA insertion in the fourth exon of *OsphyA.* The *osphyB-2* mutant is also a null allele produced by inserting T-DNA in the third intron of *OsphyB* (Jeong et al. [2007]). Afterward, F2 segregants were genotyped by PCR (35 cycles of 95°C for 15 s, 55°C for 30 s, and 72°C for 60 s), using a combination of gene-specific primers and the T-DNA primer 5′-TTGGGGTTTCTACAGGACGTAAC-3′. Those gene-specific primers were 5′-CAGGGAAAAGGGATTAGAGT-3′ and 5′-AGTGGACTCGGGTTAACTTT-3′ for *osphyA-2*, and 5′-AGTTAAGACGGAGCGACATA-3′ and 5′-GTAAGCGATCAGTTTGTGGT-3′ for *osphyB-2*. For further analyses, we selected F2 progeny that had homozygous mutant alleles for both genes.

### Vector construction and transformation

The full-length cDNA clones of *SNB* and *OsIDS1* were isolated by PCR, using primers SNB-FL-F and SNB-FL-R for *SNB*, and OsIDS1-FL-F and OsIDS1-FL-R for *OsIDS1* (Additional file 2: Table S1). After the amplified fragments were digested with *Xba* I and *Xho* I, they were inserted into the pGA3780 binary vector between the rice *actin* promoter (P*act*) and the *nopaline synthase* terminator (*nos* T) (Lee et al. [1999]; Kim et al. [2009]). For constructing a *miR172*-resistant form of *SNB* (r*SNB*), we introduced synonymous mutations into the *miR172* binding site through site-directed mutagenesis, using primer sets rSNB-N-F/rSNB-N-R and rSNB-C-F/rSNB-C-R (Additional file 2: Table S1). Each amplified fragment was digested with *Xba* I/*ACC* III or *ACC* III/*Xho* I. Afterward, the digested fragments were inserted into the pGA3780 binary vector between the rice P*act* and *nos* T. Similarly, a *miR172*-resistant form of *OsIDS1* (r*OsIDS1*) was produced using the primer sets of rOsIDS1-N-F/rOsIDS1-N-R and rOsIDS1-C-F/rOsIDS1-C-R (Additional file 2: Table S1). To obtain double-overexpression plants of r*SNB* and *Ehd1*, we amplified their full-length cDNA clones with primer sets rSNB-FL-F/rSNB-FL-R and Ehd1-FL-F/Ehd1-FL-R (Additional file 2: Table S1). The r*SNB* fragment was digested with *Bsi* WI and *Bsr* GI, and the *Ehd1* fragment was digested with *Mlu* I and *Hpa* I. Afterward, these digested fragments were inserted into the pGA3777 binary vector, a double expression cassette (Kim et al. [2009]). We have previously described plants over-expressing *miR172d* (Lee and An [2012]). The binary constructs were transformed into *Agrobacterium tumefaciens* LBA4404 (An et al. [1989]) and transgenic plants were generated via *Agrobacterium*-mediated co-cultivation (Jeon et al. [1999]).

### RNA extraction and quantitative real-time RT-PCR

Total RNA was isolated with RNAiso Plus (Takara, Shiga, Japan) and first-strand cDNA was synthesized using Moloney murine leukemia virus (M-MLV) reverse transcriptase (Promega, Madison, WI, USA) as previously reported (Ryu et al. [2009]; Lee et al. [2010]; Yang et al. [2013]). Synthesized cDNAs were used as a template for quantitative real-time RT-PCR (qRT-PCR) with SYBR® Premix Ex Taq™ II (Takara, Shiga, Japan) and the Rotor-Gene 6000 (Corbett Research, Sydney, Australia). *Osubi1* served to normalize the quantity of cDNA. All experiments were conducted at least three times, with three or more samples at each point. Primer sequences for qRT-PCR are shown in Additional file 2: Table S1. Changes in expression were calculated via the ΔΔ_Ct_ method. To ensure primer specificity, we performed the experiments when the melting curve showed a single peak. PCR products were sequenced to verify the specificity of the reaction.

### Expression analysis of microRNA

For miRNA gel blot analysis, total RNA samples were extracted from plant materials using RNAiso Plus (Takara), with a few modifications as reported previously (Jung et al. [2007]). After isopropanol precipitation, the Eppendorf tube containing the RNA pellets was briefly centrifuged without rinsing with ethanol to improve both the yield and solubility of miRNA in total RNA preparations. RNA gel blot analyses were performed with 5 μg of total RNA. Locked nucleic acid (LNA) 5′-ATgCAgCAtCAtCAaGAtTCT-3′ (upper- and lower-case letters indicate DNA and LNA, respectively) was used as an antisense oligonucleotide probe for miR172 (Varallyay et al. [2008]). The miR172 LNA probe was either labeled with P^32^ or 3′-end-labeled with DIG-ddUTP (Roche, Mannheim, Germany). Levels of the primary *miR172* (pri-*miR172*) transcripts were measured by qRT-PCR using the primer pairs described in Additional file 2: Table S1.

## Authors′ contributions

YSL and DYL designed the project, produced plant materials, monitored expression profiling, observed flowering time, analyzed data and wrote the manuscript. LHC produced Ehd1 OX plants and observed flowering time of those. GA provided an overall direction for this project and helped with the organization and editing of the manuscript. All authors read and approved the final manuscript.

## Additional files

## Electronic supplementary material

Additional file 1: Figure S1.: Temporal expressions of AP2 genes and *Ehd1* under SD. **Figure S2.** Construction of *SNB* and *OsIDS1* over-expression plants. **Figure S3.** Expressions of floral regulators in the *OsIDS1* OX. **Figure S4.** Expressions of floral regulators in the *SNB* OX. **Figure S5.** Expression levels of AP2s in the various flowering-time mutants. **Figure S6.** Phenotypes of *miR172*-resistant *OsIDS1* overexpression. **Figure S7.** Verification of r*SNB* construct. **Figure S8.** Phenotypes of *Ehd1* overexpression. (PDF 415 KB)

Additional file 2: Table S1.: Sequences of primers used in this study. (PDF 61 KB)

Below are the links to the authors’ original submitted files for images.

Authors’ original file for figure 1

Authors’ original file for figure 2

Authors’ original file for figure 3

Authors’ original file for figure 4

Authors’ original file for figure 5

Authors’ original file for figure 6

Authors’ original file for figure 7

Authors’ original file for figure 8
