# Supplementary material for: Rice miR172 induces flowering by suppressing OsIDS1 and SNB, two AP2 genes that negatively regulate expression of Ehd1 and florigens
Source: Rice (N Y). 2014 Nov 19;7:31. doi: 10.1186/s12284-014-0031-4 (PMC4884018; doi:10.1186/s12284-014-0031-4)
Supplement: Supplementary file 1 — Additional file 1: Figure S1.: Temporal expressions of AP2 genes and Ehd1 under SD. Figure S2. Construction of SNB and OsIDS1 over-expression plants. Figure S3. Expressions of floral regulators in the OsIDS1 OX. Figure S4. Expressions of floral regulators in the SNB OX. Figure S5. Expression levels of AP2s in the various flowering-time mutants. Figure S6. Phenotypes of miR172-resistant OsIDS1 overexpression. Figure S7. Verification of rSNB construct. Figure S8. Phenotypes of Ehd1 overexpression. (PDF 415 KB) [file 12284_2014_31_MOESM1_ESM.pdf]

Additional files 1:

Figure S1. Temporal expressions of AP2 genes and *Ehd1* under SD.

(A,B) RT-PCR analysis of the transcript levels of *SNB* (A) and *OsIDS1* (B) under SD. Middle regions of fully emerged uppermost leaves were harvested at 15, 18, 21, 24, 27 and 30 DAG. (C) RT-PCR analysis of the transcript levels of *Ehd1* under SD. Middle regions of fully emerged uppermost leaves were harvested at 15, 18, 21, 24, 27 and 30 DAG. Expressions of the AP2 genes and *Ehd1* were analyzed at ZT 2 h. Y axis, relative transcript level compared with rice *ubi1*. Error bars indicate standard deviations; n = 4 or more.

Figure S2. Construction of *SNB* and *OsIDS1* over-expression plants.

(A-C) Expression levels of *SNB* (endogenous) and t*SNB* (transgene) in the *SNB* OX plants via RT-PCR (A) and qRT-PCR (B, C). *Ubiquitin1* was used as reference.(D-F) Expression levels of *OsIDS1* (endogenous) and t*OsIDS1* (transgene) in the *OsIDS1* OX plants via RT-PCR (D) and qRT-PCR (E, F). *Ubiquitin1* was used as reference.

Figure S3. Expressions of floral regulators in the *OsIDS1* OX.

(A-H) Expression levels of *OsPhyB* (A), *OsMADS50* (B), *Ghd7* (C), *OsId1* (D), *OsCOL4* (E), *OsMADS56* (F), *Ehd3* (G) and *OsTrx1* (H) in the wild-type (closed boxes) and *OsIDS1* overexpression plants (open boxes) under short day (SD) conditions. For expression analysis, plants were grown in SD (12 h light/ 12 h dark) conditions. Middle regions of fully emerged uppermost leaves were harvested at 30 days after germination. Expressions of these genes were monitored at ZT 2 h. Y axis, relative transcript level of each gene compared with that of rice *ubi1*. Error bars indicate standard deviations; *n* = 4 or more.

Figure S4. Expressions of floral regulators in the *SNB* OX.

(A-H) Expression levels of *SNB* (A), *Hd3a* (B), *RFT1* (C), *Ehd1* (D), *OsGI* (E), *Hd1* (F), *OsMADS51* (G) and *OsCOL4* (H) in the wild-type (closed boxes) and *SNB* over-expression plants (open boxes) under short day (SD) conditions. For expression analysis, plants were grown in SD (12 h light/ 12 h dark) conditions. Middle regions of fully emerged uppermost leaves were harvested at 30 days after germination. Expressions of *OsGI* and *Hd1* were observed at ZT 10 h and ZT 14 h, respectively and other regulators were monitored at ZT 2 h. Y axis, relative transcript level of each gene compared with that of rice *ubi1*. Error bars indicate standard deviations; *n* = 4 or more.

Figure S5. Expression levels of AP2s in the various flowering-time mutants.

(A, B) Expression levels of *SNB* (A) and *OsIDS1* (B) in the Dongjin (black bars), Kitaake (gray bars), *ghd7*, *osgi*, *Ehd1* RNAi, *hd1*, *osmads50*, *osmads51*, *OsId1* RNAi, *ostrx1* and *osvil2* (open bars) plants. For expression analysis, middle regions of fully emerged uppermost leaves were harvested at 40 days after germination. Expressions of AP2 genes were observed at ZT 2 h. Y axis, relative transcript level of each gene compared with that of rice *ubi1*. Expressions of AP2 genes in each mutant were normalized by those of WT. Error bars indicate standard deviations; *n* = 4 or more.

Figure S6. Phenotypes of *miR172*-resistant *OsIDS1*overexpression.

(A) Scheme of *miR172*-resistant *OsIDS1* (r*OsIDS1*) construct. Four silent mutations (red characters) were introduced into *miR172* target sequences on C-terminus of *OsIDS1* full-length cDNA. Primers t*OsIDS1* F and R were used for observing a transgene expression of *OsIDS1* in the r*OsIDS1* over-expression plants. Underlined and italic sequences (*tccgga*) were indicated restriction enzyme site by *Aor13*HI or *ACC*III. A.A, Amino Acid sequences; *nos*T, *nos* terminator; P*act*, *actin* promoter. (B) Expression level of *OsIDS1* (transgene) in the r*OsIDS1* OX plants. (C) Phenotypes of r*OsIDS1* OX plants. Numbers indicated individual primary transgenic number of r*OsIDS1* OX plants. Plants were grown under short day conditions (12 h light/ 12 h dark). Photograph was taken at 132 DAT (days after transfer of regenerates).

Figure S7. Verification of r*SNB* construct

(A) Restriction enzyme site for *Aor13*HI and *ACC*III in the r*SNB* construct.

(B) Digestion of t*SNB* PCR products for the *SNB* and r*SNB* by *ACC*III.

Figure S8. Phenotypes of *Ehd1*overexpression.

(A) Scheme of *Ehd1* OX construct. *Ehd1* full-length cDNA was sub-cloned into pGA3426 vector between maize *ubiquitin* promoter (P*ubi*) and *nopaline synthase* terminator (*nos*T). (B) Phenotypes of *Ehd1* OX plants. Plants were grown under short day conditions (12 h light/ 12 h dark). Photograph was taken at 42 DAT (days after transfer of regenerates). Red arrows indicate emerged panicles.

Figure S1

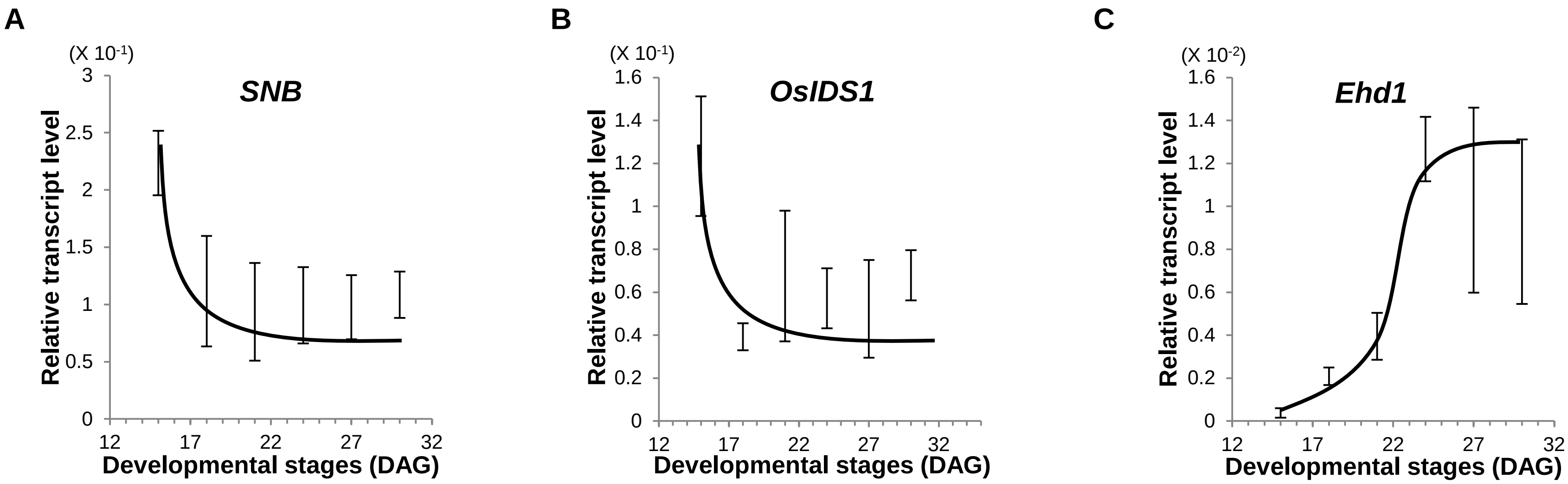

Figure S1. Temporal expressions of AP2 genes and *Ehd1* under SD.

(A,B) RT-PCR analysis of the transcript levels of *SNB* (A) and *OsIDS1* (B) under SD. Middle regions of fully emerged uppermost leaves were harvested at 15, 18, 21, 24, 27 and 30 DAG. (C) RT-PCR analysis of the transcript levels of *Ehd1* under SD. Middle regions of fully emerged uppermost leaves were harvested at 15, 18, 21, 24, 27 and 30 DAG. Expressions of the AP2 genes and *Ehd1* were analyzed at ZT 2 h. Y axis, relative transcript level compared with rice *ubi1*. Error bars indicate standard deviations; n = 4 or more.

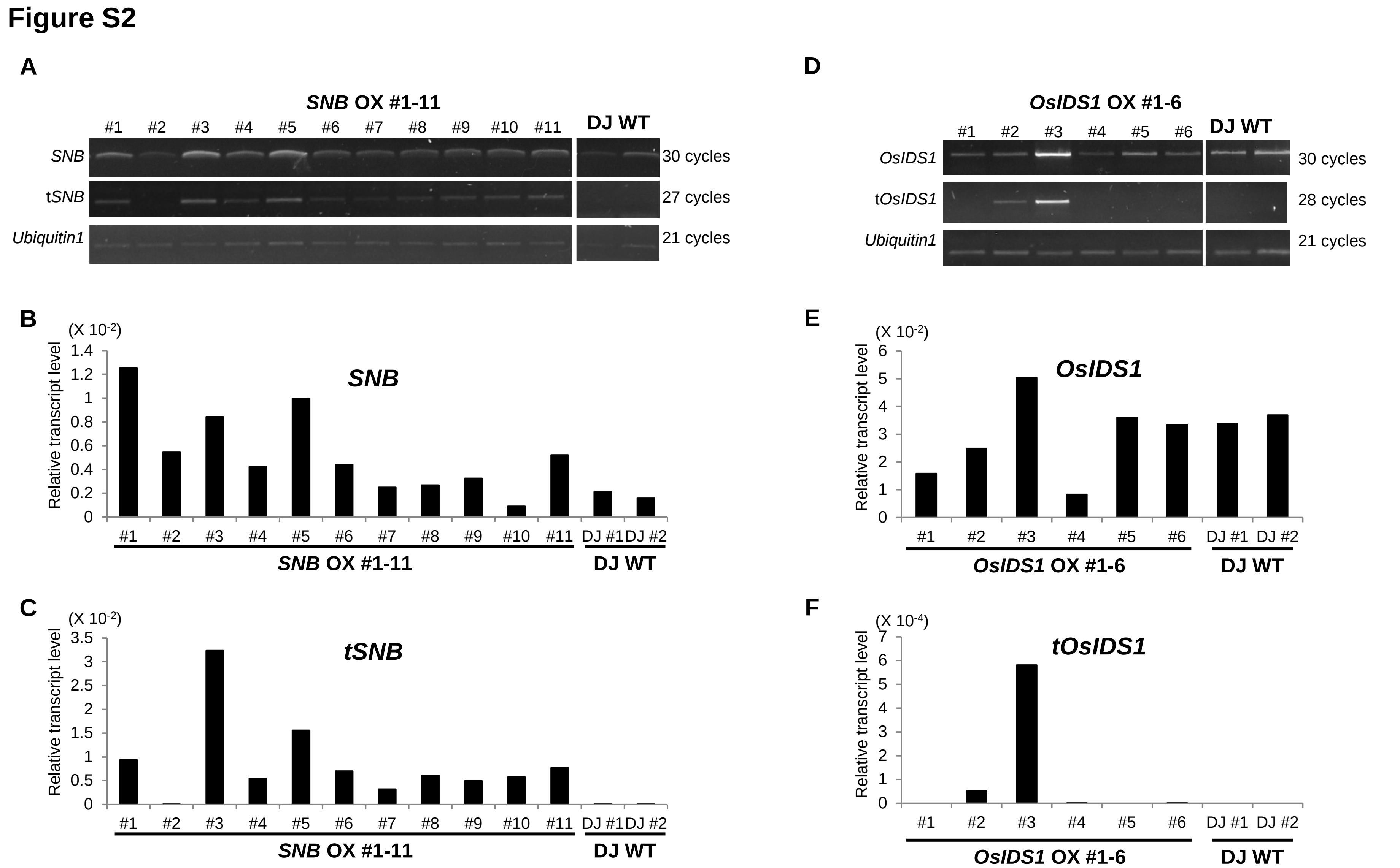

Figure S2. Construction of *SNB* and *OsIDS1* over-expression plants.

(A-C) Expression levels of *SNB* (endogenous) and t*SNB* (transgene) in the *SNB* OX plants via RT-PCR (A) and qRT-PCR (B, C). *Ubiquitin1* was used as reference.

(D-F) Expression levels of *OsIDS1* (endogenous) and t*OsIDS1* (transgene) in the *OsIDS1* OX plants via RT-PCR (D) and qRT-PCR (E, F). *Ubiquitin1* was used as reference.

Figure S3

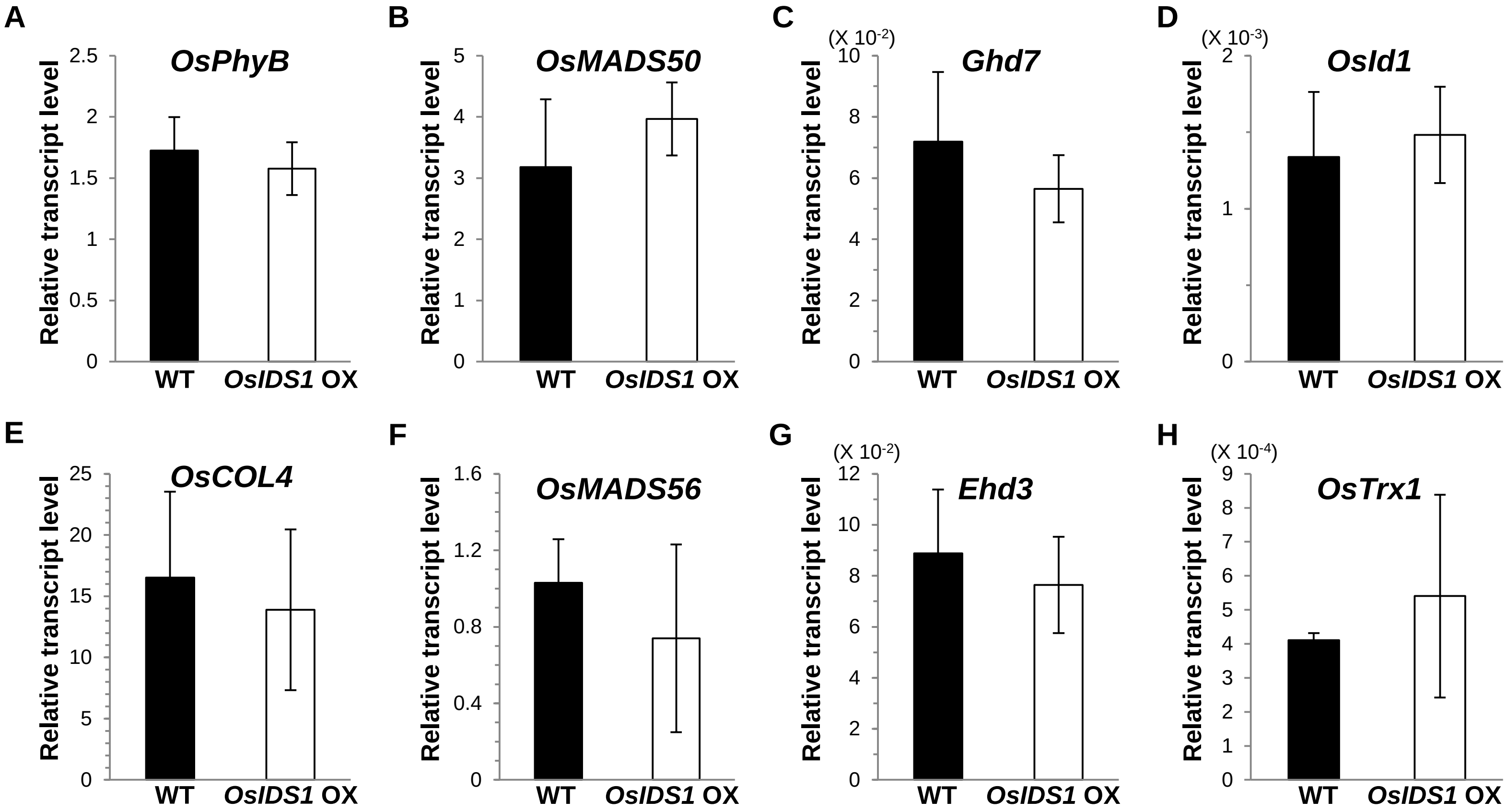

Figure S3. Expressions of floral regulators in the *OsIDS1* OX.

(A-H) Expression levels of *OsPhyB* (A), *OsMADS50* (B), *Ghd7* (C), *OsId1* (D), *OsCOL4* (E), *OsMADS56* (F), *Ehd3* (G) and *OsTrx1* (H) in the wild-type (closed boxes) and *OsIDS1* overexpression plants (open boxes) under short day (SD) conditions. For expression analysis, plants were grown in SD (12 h light/ 12 h dark) conditions. Middle regions of fully emerged uppermost leaves were harvested at 30 days after germination. Expressions of these genes were monitored at ZT 2 h. Y axis, relative transcript level of each gene compared with that of rice *ubi1*. Error bars indicate standard deviations; n = 4 or more.

Figure S4

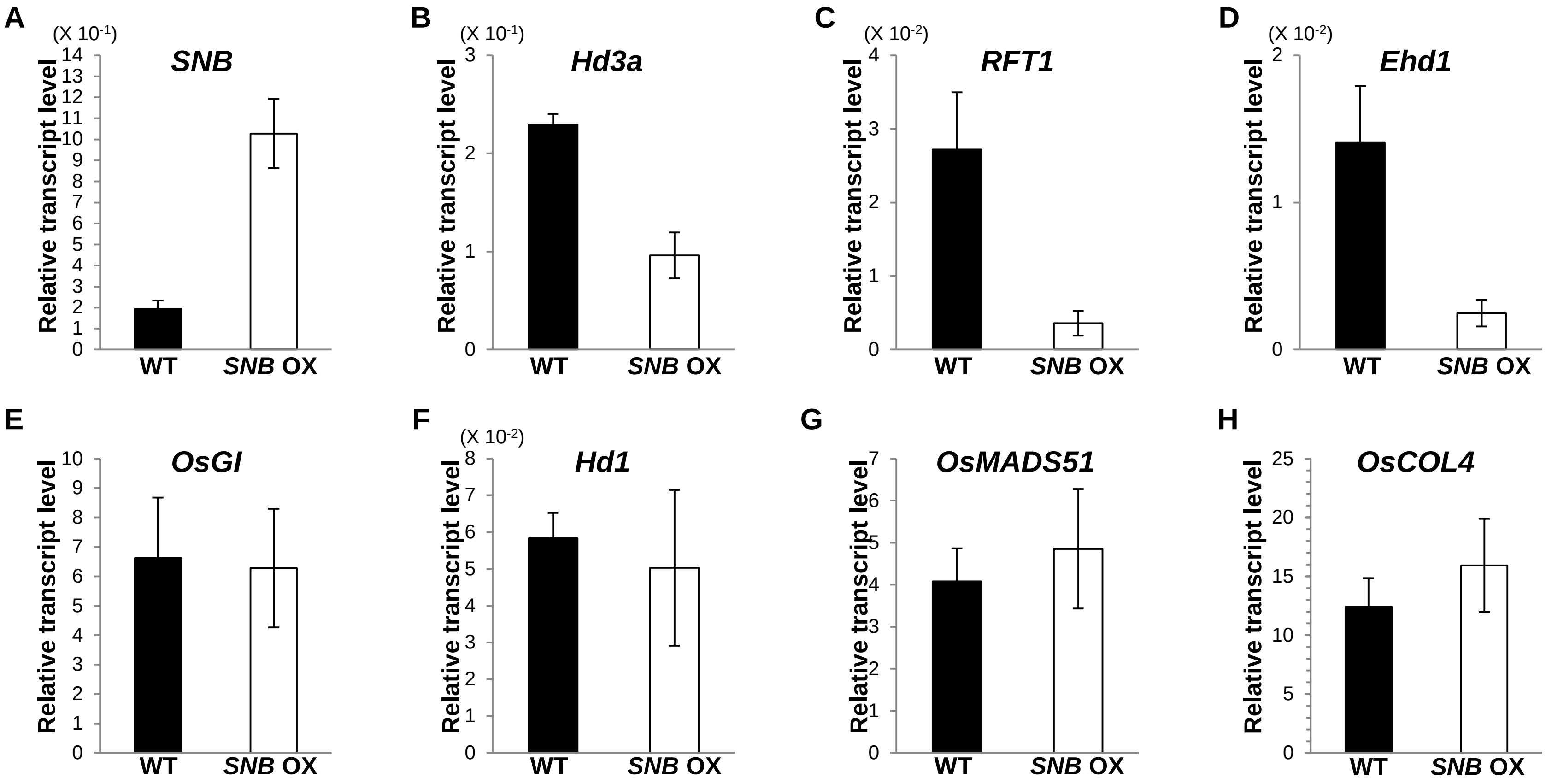

Figure S4. Expressions of floral regulators in the *SNB* OX.

(A-H) Expression levels of *SNB* (A), *Hd3a* (B), *RFT1* (C), *Ehd1* (D), *OsGI* (E), *Hd1* (F), *OsMADS51* (G) and *OsCOL4* (H) in the wild-type (closed boxes) and *SNB* over-expression plants (open boxes) under short day (SD) conditions. For expression analysis, plants were grown in SD (12 h light/ 12 h dark) conditions. Middle regions of fully emerged uppermost leaves were harvested at 30 days after germination. Expressions of *OsGI* and *Hd1* were observed at ZT 10 h and ZT 14 h, respectively and other regulators were monitored at ZT 2 h. Y axis, relative transcript level of each gene compared with that of rice *ubi1*. Error bars indicate standard deviations; *n* = 4 or more.

Figure S5

A

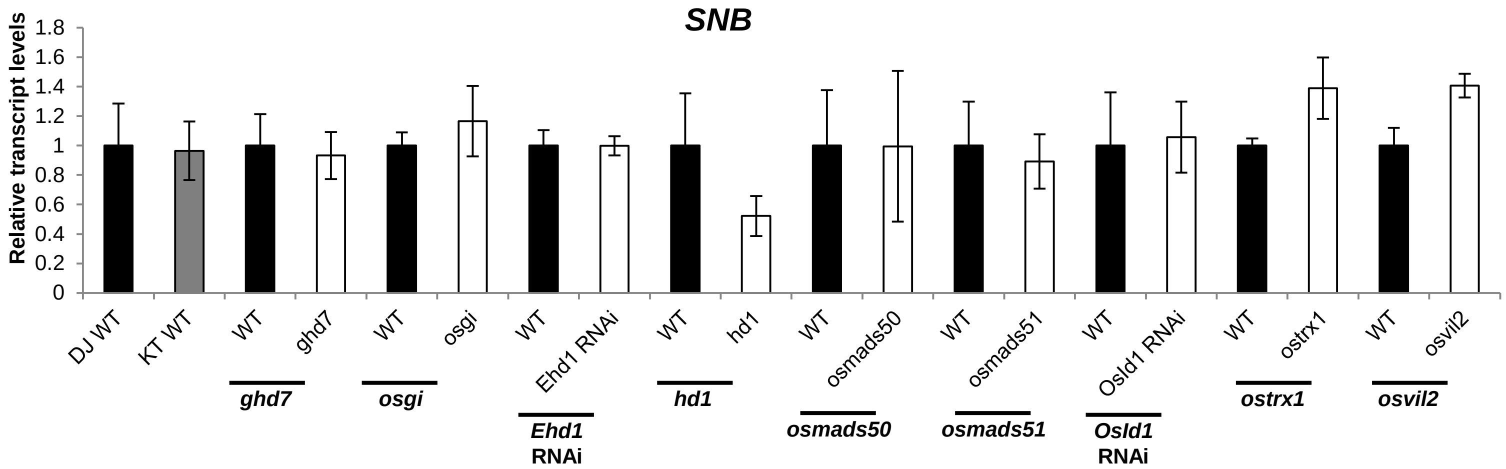

B

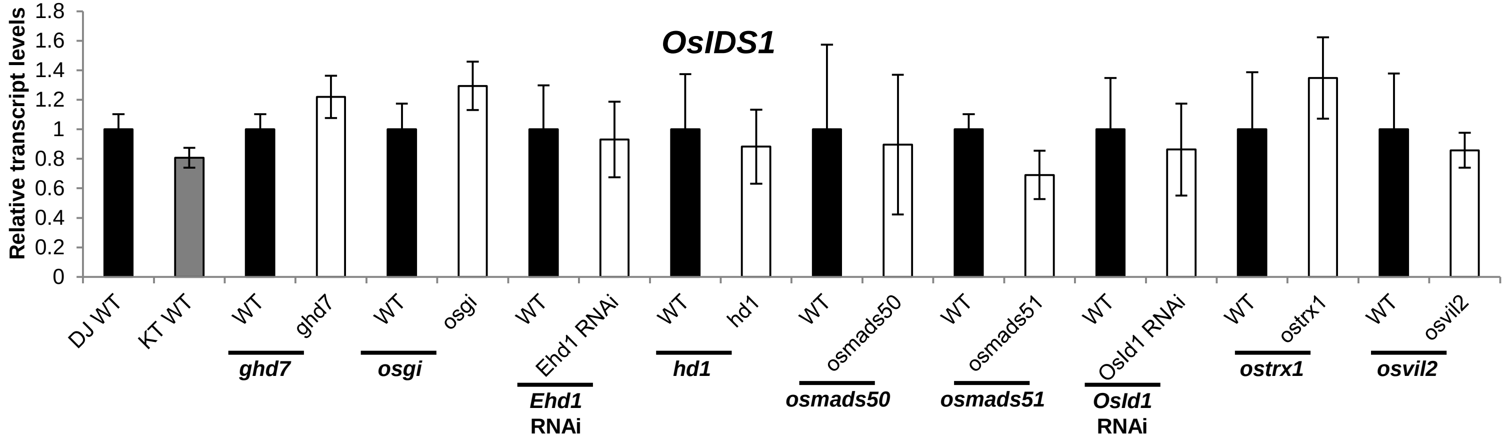

Figure S5. Expression levels of AP2s in the various flowering-time mutants.

(A, B) Expression levels of *SNB* (A) and *OsIDS1* (B) in the Dongjin (black bars), Kitaake (gray bars), *ghd7*, *osgi*, *Ehd1* RNAi, *hd1*, *osmads50*, *osmads51*, *OsId1* RNAi, *ostrx1* and *osvil2* (open bars) plants. For expression analysis, middle regions of fully emerged uppermost leaves were harvested at 40 days after germination. Expressions of AP2 genes were observed at ZT 2 h. Y axis, relative transcript level of each gene compared with that of rice *ubi1*. Expressions of AP2 genes in each mutant were normalized by those of WT. Error bars indicate standard deviations; $n = 4$ or more.

Figure S6

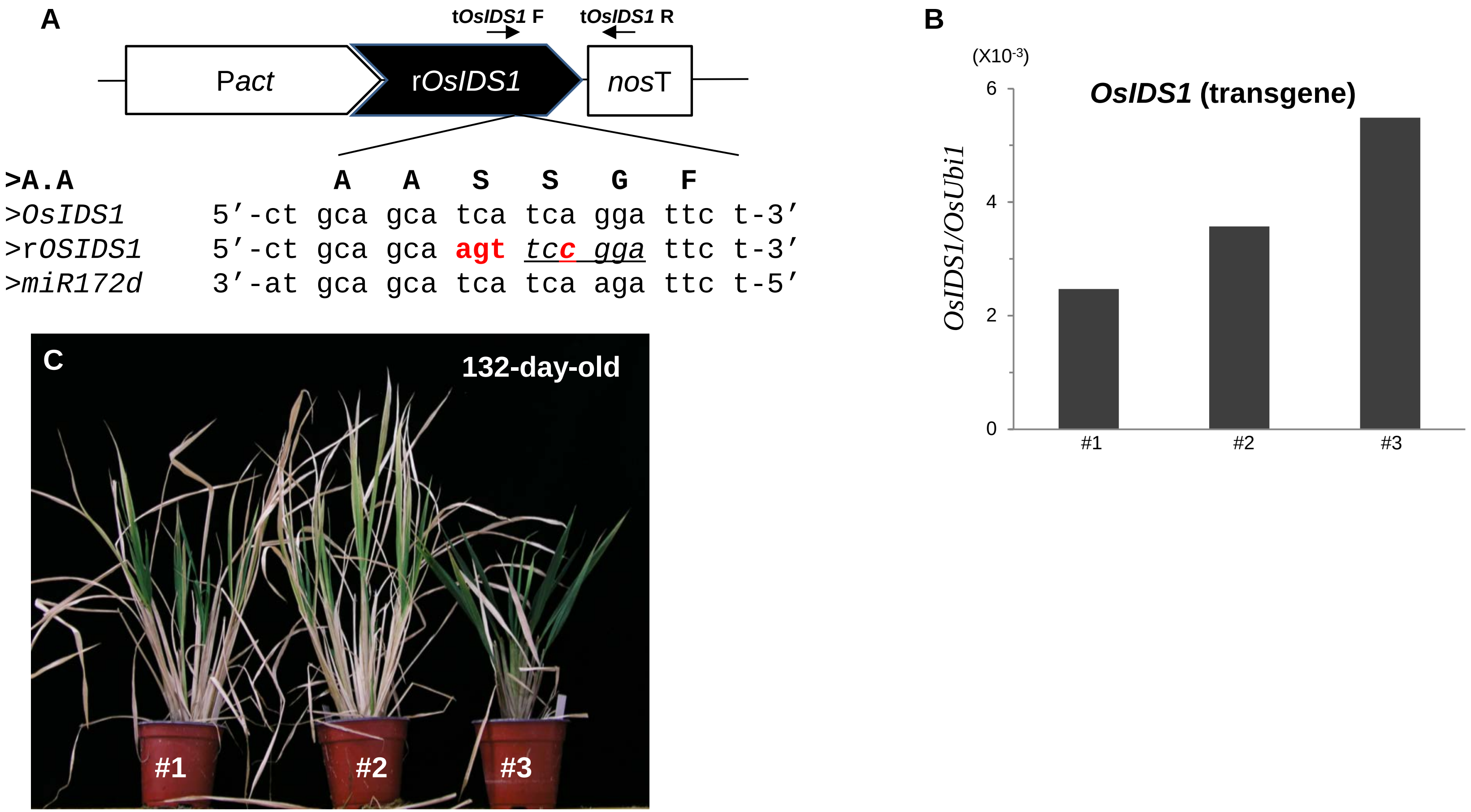

Figure S6. Phenotypes of *miR172*-resistant *OsIDS1*overexpression.

(A) Scheme of *miR172*-resistant *OsIDS1* (r*OsIDS1*) construct. Four silent mutations (red characters) were introduced into *miR172* target sequences on C-terminus of *OsIDS1* full-length cDNA. Primers t*OsIDS1* F and R were used for observing a transgene expression of *OsIDS1* in the r*OsIDS1* over-expression plants. Underlined and italic sequences (*tccgga*) were indicated restriction enzyme site by *Aor13*HI or *ACC*III. A.A, Amino Acid sequences; *nos*T, *nos* terminator; P*act*, *actin* promoter. (B) Expression level of *OsIDS1* (transgene) in the r*OsIDS1* OX plants. (C) Phenotypes of r*OsIDS1* OX plants. Numbers indicated individual primary transgenic number of r*OsIDS1* OX plants. Plants were grown under short day conditions (12 h light/ 12 h dark). Photograph was taken at 132 DAT (days after transfer of regenerates).

Figure S7

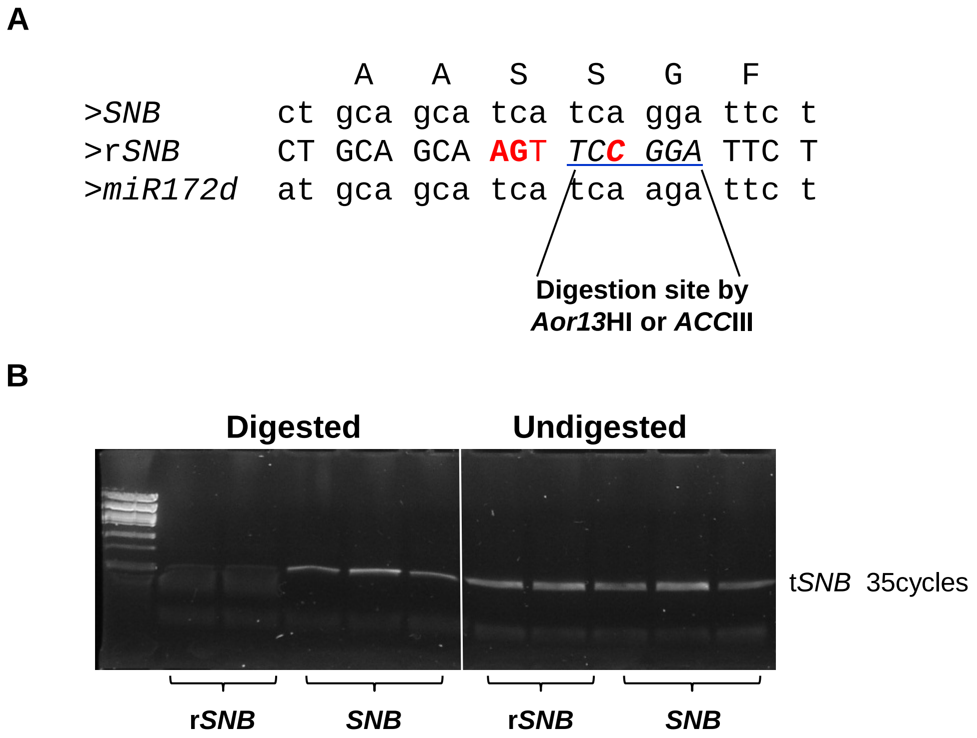

Figure S7. Verification of r*SNB* construct

(A) Restriction enzyme site for *Aor13*HI and *ACC*III in the r*SNB* construct.

(B) Digestion of t*SNB* PCR products for the *SNB* and r*SNB* by *ACC*III.

Figure S8

A

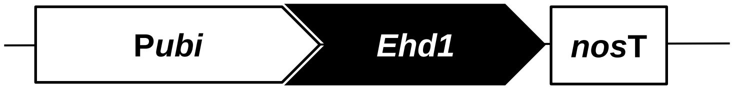

B

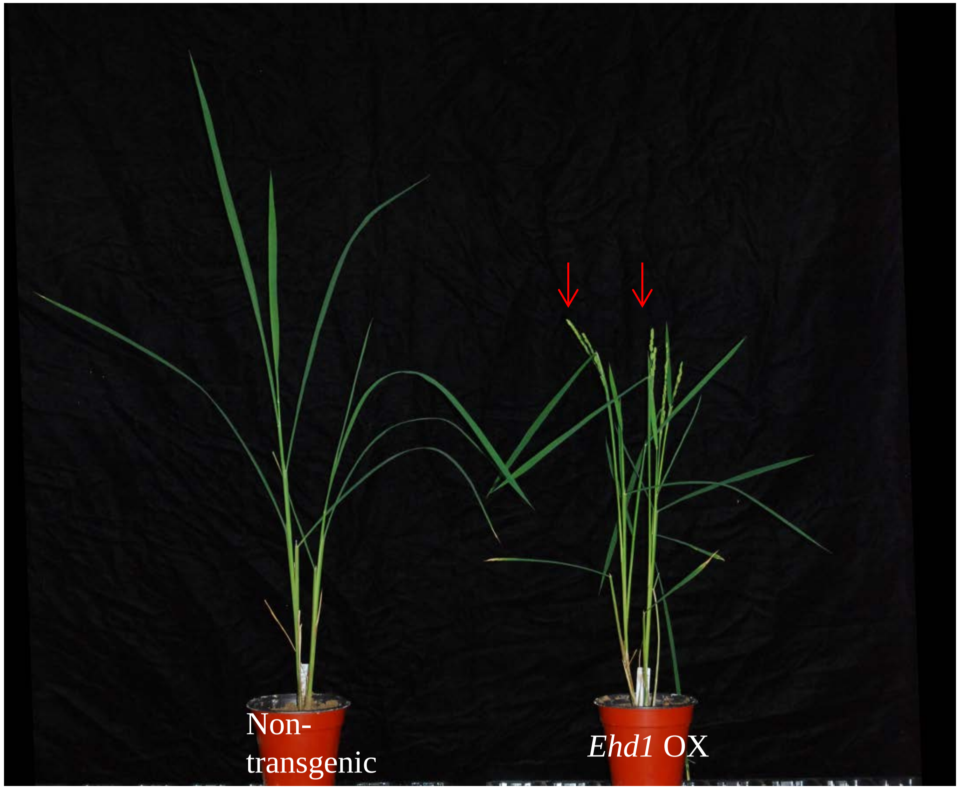

Figure S8. Phenotypes of *Ehd1*overexpression.

(A) Scheme of *Ehd1* OX construct. *Ehd1* full-length cDNA was sub-cloned into pGA3426 vector between maize *ubiquitin* promoter (P*ubi*) and *nopaline synthase* terminator (*nos*T). (B) Phenotypes of *Ehd1* OX plants. Plants were grown under short day conditions (12 h light/ 12 h dark). Photograph was taken at 42 DAT (days after transfer of regenerates). Red arrows indicate emerged panicles.
