# Supplementary material for: Rice miR172 induces flowering by suppressing OsIDS1 and SNB, two AP2 genes that negatively regulate expression of Ehd1 and florigens
Source: Rice (N Y). 2014 Nov 19;7:31. doi: 10.1186/s12284-014-0031-4 (PMC4884018; doi:10.1186/s12284-014-0031-4)
Supplement: Supplementary file 2 — Additional file 2: Table S1.: Sequences of primers used in this study. (PDF 61 KB) [file 12284_2014_31_MOESM2_ESM.pdf]

Additional files 2:

Table S1. Sequences of primers used in this study

Table S1. Sequences of primers used in this study

Primer name	Gene	Sequence
**Primers for qRT-PCR**		
Ubi-RT-F	*Ubiquitin1*	5'-AACCAGCTGAGGCCCAAGA-3'
Ubi-RT-R		5'-ACGATTGATTTAACCAGTCCATGA-3'
pri-miR172a-RT-F	*miR172a*	5'-CGTGGCATCATCAAGATTCACATC-3'
pri-miR172a-RT-R		5'-CAAGATTCTCAGCCAAATCGGATG-3'
pri-miR172d-RT-F	*miR172d*	5'-ACAGTCGGTGCTTGCAGGTG-3'
pri-miR172d-RT-R		5'-TCACATATAGTCAGCCAACCTCG-3'
SNB-RT-F	*SNB*	5'-ATGGAAGGGAAGCTGTTAC-3'
SNB-RT-R		5'-AATGTGGATGCTGGGACATC-3'
OsIDS1-RT-F	*OsIDS1*	5'-CTGGCCTCCAGTTAACTTGT-3'
OsIDS1-RT-R		5'-GGCGCCGGCAGAGAATCCT-3'
tSNB-RT-F	t*SNB*	5'-GTCCCCAGTCGTTCCCTACT-3'
tSNB-RT-R		5'-CCATCTCATAAATAACGTCATGC-3'
tOsIDS1-RT-F	t*OsIDS1*	5'-ACTGCCCCCTCAGCATCAAC-3'
tOsIDS1-RT-R		5'-GAACTTCAGGGTCAGCTTGCC-3'
OsTOE1-RT-F	*OsTOE1*	5'-GATGCATCCAGATCATCGGA-3'
OsTOE1-RT-R		5'-AGACTAGGAGCATTTGGAAC-3'
OsGI-RT-F	*OsGI*	5'-ATCGTTCTGCAGGCCGAGA-3'
OsGI-RT-R		5'-TCACCAATGCTTCTGGGCTAT-3'
Hd1-RT-F	*Hd1*	5'-TCAGCAACAGCATATCTTTCTCATCA-3'
Hd1-RT-R		5'-TCTGGAATTTGGCATATCTATCACC-3'
Ehd1-RT-F	*Ehd1*	5'-TGCAAATGGCGCTTTTGAT-3'
Ehd1-RT-R		5'-ATATGTGCTGCCAAATGTTGCT-3'
Hd3a-RT-F	*Hd3a*	5'-GCTCACTATCATCATCCAGCATG-3'
Hd3a-RT-R		5'-CCTTGCTCAGCTATTTAATTGCATAA-3'
RFT1-RT-F	*RFT1*	5'-TGACCTAGATTCAAAGTCTAATCCTT-3'
RFT1-RT-R		5'-TGCCGGCCATGTCAAATTAATAAC-3'
OsPhyB-RT-F	*OsPhyB*	5'-ATGGAACAGACACAATGCTT-3'
OsPhyB-RT-R		5'-AGCATACACCATATCAGCTT-3'
Ghd7-RT-F	*Ghd7*	5'-ATATTGTGGGAGCACGTT-3'
Ghd7-RT-R		5'-ATCTGAACCATTGTCCAAGC-3'
OsId1-RT-F	*OsId1*	5'-CCTCTTCTCCAGGAAGGACA -3'
OsId1-RT-R		5'-GCTGCTGGTGATCAGAAGATT-3'
OsMADS50-RT-F	*OsMADS50*	5'-AAAGCTGACGCTGATGGTTTG-3'
OsMADS50-RT-R		5'-GTTTCGACATCCATGTTGTC-3'
OsMADS51-RT-F	*OsMADS51*	5'-GTTTGCTCTGCTCCTACTC-3'
OsMADS51-RT-R		5'-ACTCCTCCTCCAGCATTGAA-3'
OsMADS56-RT-F	*OsMADS56*	5'-GACCGCTATAAAGCATACACA-3'
OsMADS56-RT-R		5'-TCATGTGGTTAGCCACCAGC-3'
OsCOL4-RT-F	*OsCOL4*	5'-ATCCACTCGGCGAACCCGCT -3'
OsCOL4-RT-R		5'-CGCTTCTCCCTGTACCGCAT-3'
OsCO3-RT-F	*OsCO3*	5'-GGAGAAGAGGAAGACGAGGC-3'
OsCO3-RT-R		5'-TAGCTAAGCAACCAAGATGTA-3'
Ehd3-RT-F	*Ehd3*	5'-GGACCACCTCGTCACCTACAA-3'
Ehd3-RT-R		5'-CGCCGTTGGCCATGAG-3'
OsTrx1-RT-F	*OsTrx1*	5'-GGTCACATCAGAAGATGGAA-3'

OsTrx1-RT-R		5'-CACCATAGCATCTAGCATGTA-3'
OsVIL2-RT-F	*OsVIL2*	5'-CGGAAACAGAATGATCTGCTT-3'
OsVIL2-RT-R		5'-GAAGTTTGATGGTATCATCCG-3'
**Primers for cloning**		
SNB-FL-F	*SNB*	5'-gctctagaATGGTGCTGGAT-3'
SNB-FL-R		5'-tctcgagGGCGGTCGGGGGA-3'
OsIDS1-FL-F	*OsIDS1*	5'-ctctagaATGTTGTTGGATCT-3'
OsIDS1-FL-R		5'-tctcgagGGCGGTTGGCGGGA-3'
rSNB-N-F	r*SNB*	5'-gctctagaATGGTGCTGGAT-3'
rSNB-N-R		5'-GTAGAGAAtccggaACTTGCTGCAGTAG-3'
rSNB-C-F		5'-CTACTGCAGCAAGTtccggaTTCTCTAC-3'
rSNB-C-R		5'-tctcgagGGCGGTCGGGGGA-3'
rOsIDS1-N-F	r*OsIDS1*	5'-ctctagaATGTTGTTGGATCT-3'
rOsIDS1-N-R		5'-CAGAGAAtccggaACTTGCTGCAGCG-3'
rOsIDS1-C-F		5'-CGCTGCAGCAAGTtccggaTTCTCTG-3'
rOsIDS1-C-R		5'-tctcgagGGCGGTTGGCGGGA-3'
rSNB-FL-F	r*SNB*	5'-cgtacgATGGTGCTGGATCTCA-3'
rSNB-FL-R		5'-tgtacaTCAGGCGGTCGGGG-3'
Ehd1-FL-F	*Ehd1*	5'-acgcgtATGGATCACCGAGAGC-3'
Ehd1-FL-R		5'-gttaacCTAGAAATTCCAAAAAC-3'
