# Supplementary figures and images for: Rice miR172 induces flowering by suppressing OsIDS1 and SNB, two AP2 genes that negatively regulate expression of Ehd1 and florigens
Source: Rice (N Y). 2014 Nov 19;7:31. doi: 10.1186/s12284-014-0031-4 (PMC4884018; doi:10.1186/s12284-014-0031-4)

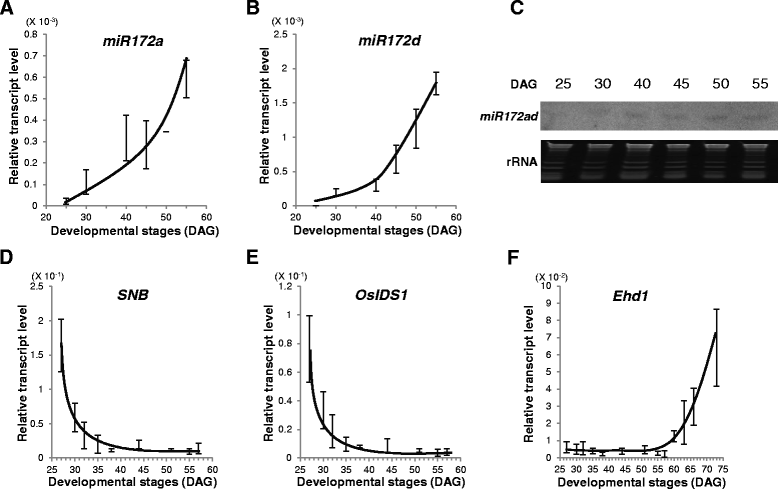

Supplement: Supplementary file 3 — Authors’ original file for figure 1 [file 12284_2014_31_MOESM3_ESM.gif]

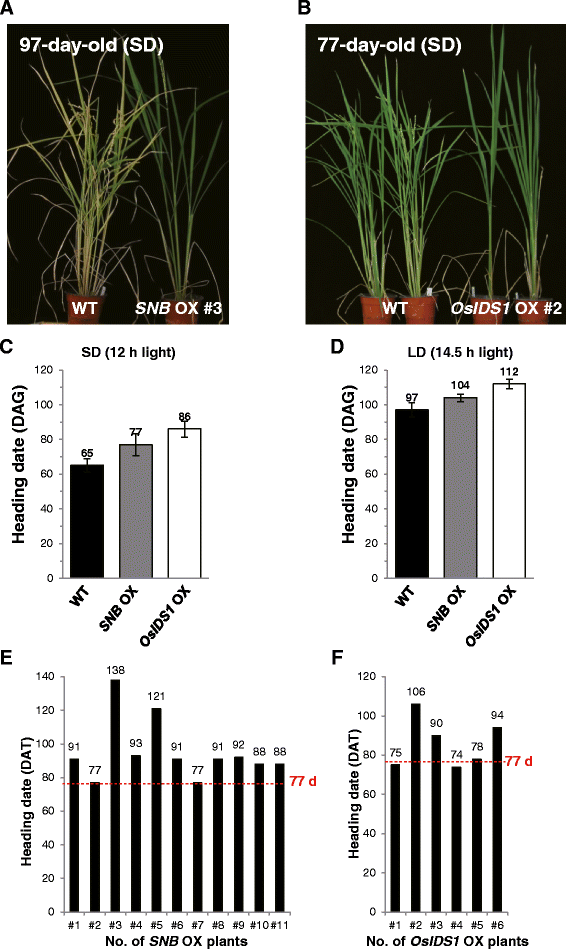

Supplement: Supplementary file 4 — Authors’ original file for figure 2 [file 12284_2014_31_MOESM4_ESM.gif]

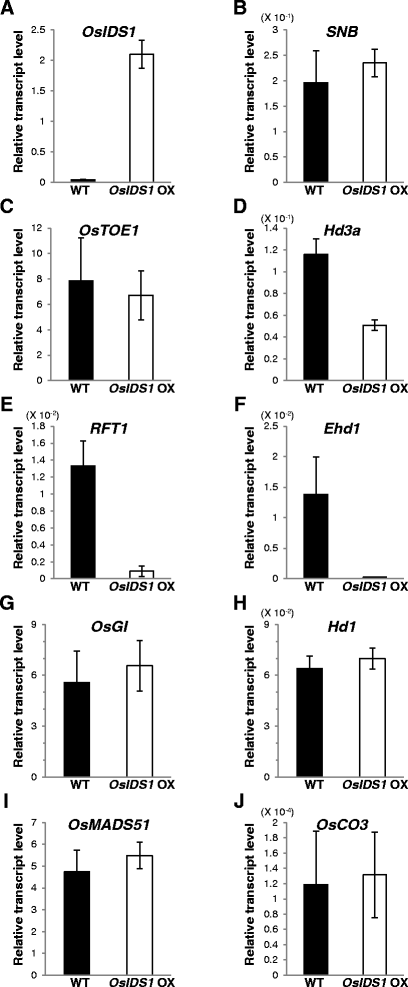

Supplement: Supplementary file 5 — Authors’ original file for figure 3 [file 12284_2014_31_MOESM5_ESM.gif]

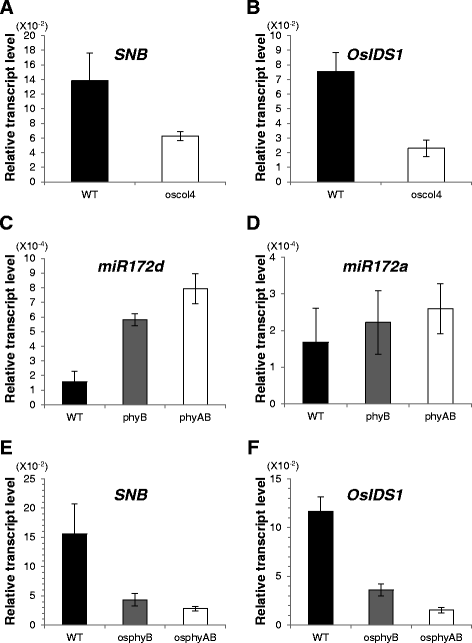

Supplement: Supplementary file 6 — Authors’ original file for figure 4 [file 12284_2014_31_MOESM6_ESM.gif]

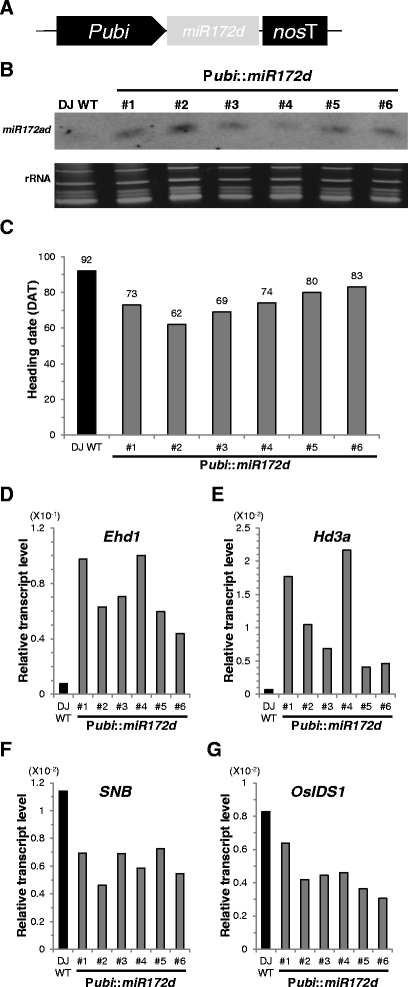

Supplement: Supplementary file 7 — Authors’ original file for figure 5 [file 12284_2014_31_MOESM7_ESM.gif]

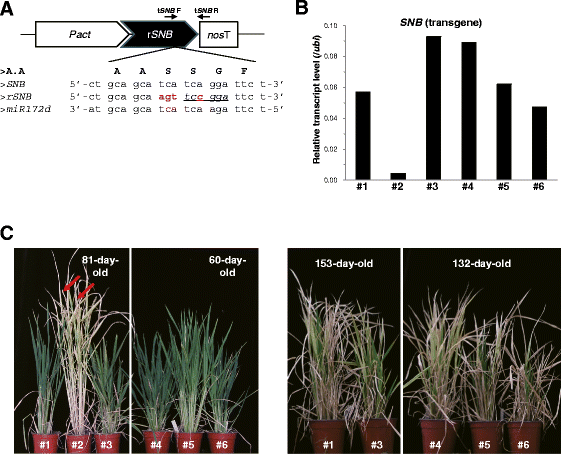

Supplement: Supplementary file 8 — Authors’ original file for figure 6 [file 12284_2014_31_MOESM8_ESM.gif]

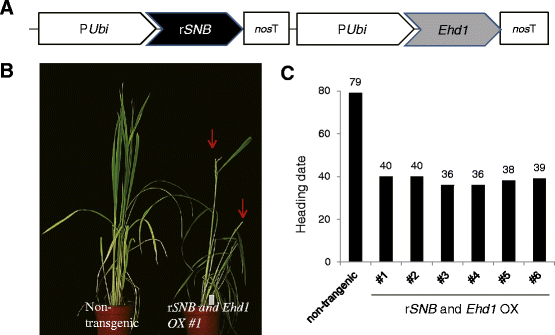

Supplement: Supplementary file 9 — Authors’ original file for figure 7 [file 12284_2014_31_MOESM9_ESM.gif]

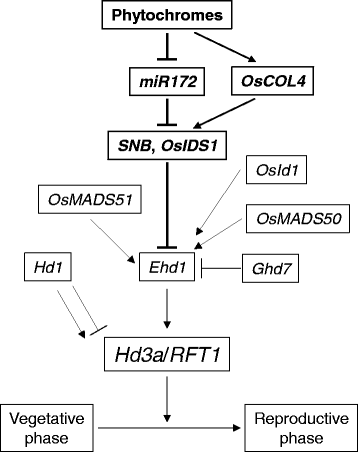

Supplement: Supplementary file 10 — Authors’ original file for figure 8 [file 12284_2014_31_MOESM10_ESM.gif]
